# Cry75Aa (Mpp75Aa) Insecticidal Proteins for Controlling the Western Corn Rootworm, Diabrotica virgifera *virgifera* LeConte (Coleoptera: Chrysomelidae), Isolated from the Insect-Pathogenic Bacterium Brevibacillus laterosporus

**DOI:** 10.1128/AEM.02507-20

**Published:** 2021-02-12

**Authors:** David Bowen, Yong Yin, Stanislaw Flasinski, Catherine Chay, Gregory Bean, Jason Milligan, William Moar, Aihong Pan, Brent Werner, Karrie Buckman, Arlene Howe, Todd Ciche, Keith Turner, Michael Pleau, Jun Zhang, Jean-Louis Kouadio, Bruce E. Hibbard, Paula Price, James Roberts

**Affiliations:** a Bayer Crop Science, Chesterfield, Missouri, USA; b USDA-ARS, Plant Genetics Research Unit, University of Missouri, Columbia, Missouri, USA; University of Queensland

**Keywords:** *Bacillus thuringiensis*, biotechnology, *Brevibacillus laterosporus*, Mpp75Aa, genetically modified plants, insect control, insecticidal proteins, western corn rootworm

## Abstract

This study describes three closely related proteins cloned from Brevibacillus laterosporus strains that are lethal upon feeding to Diabrotica virgifera virgifera LeConte, the western corn rootworm (WCR). Mpp75Aa1, Mpp75Aa2, and Mpp75Aa3 were toxic to WCR larvae when the larvae were fed purified protein. Transgenic plants expressing each mMpp75Aa protein were protected from feeding damage and showed a significant reduction in adult emergence from infested plants by both susceptible Cry3Bb1 and Cry34Ab1/Cry35Ab1-resistant WCR. These results demonstrate that proteins from B. laterosporus are as efficacious as the well-known Bacillus thuringiensis insecticidal proteins in controlling major insect pests such as WCR. The deployment of transgenic maize expressing mMpp75Aa, along with other active molecules lacking cross-resistance, has the potential to be a useful tool for control of WCR populations resistant to current B. thuringiensis traits.

**IMPORTANCE** Insects feeding on roots of crops can damage the plant roots, resulting in yield loss due to poor water and nutrient uptake and plant lodging. In maize, the western corn rootworm (WCR) can cause severe damage to the roots, resulting in significant economic loss for farmers. Genetically modified (GM) plants expressing Bacillus thuringiensis insect control proteins have provided a solution for control of these pests. In recent years, populations of WCR resistant to the B. thuringiensis proteins in commercial GM maize have emerged. There is a need to develop new insecticidal traits for the control of WCR populations resistant to current commercial traits. New proteins with commercial-level efficacy on WCR from sources other than B. thuringiensis are becoming more critical. The Mpp75Aa proteins from B. laterosporus, when expressed in maize, are efficacious against the resistant populations of WCR and have the potential to provide solutions for control of resistant WCR.

## Abstract (precis)

Insects feeding on roots of crops can damage the plant roots, resulting in yield loss due to poor water and nutrient uptake and plant lodging. In maize, the western corn rootworm (WCR) can cause severe damage to the roots, resulting in significant economic loss for farmers.

## INTRODUCTION

Genetically modified (GM) maize and cotton plants expressing Bacillus thuringiensis insecticidal proteins have been commercially available since 1995 and were quickly adopted by farmers in the United States (https://www.ers.usda.gov/data-products/adoption-of-genetically-engineered-crops-in-the-us/recent-trends-in-ge-adoption.aspx). In 2003, Cry3Bb1 became commercially available to control western corn rootworm (WCR) (Diabrotica virgifera virgifera) (1). In 2005, the binary insecticidal protein Cry34Ab1/Cry35Ab1 (assigned the new designations of Gpp34Ab1/Tpp35Ab1 by the Bacterial Pesticidal Protein Resource Center [BPPRC]) (2), with no cross-resistance to Cry3Bb1, became available for the control of WCR (3).

Field-selected resistance to Cry3Bb1 was first reported in 2009 (4), and a subsequent study in 2016 reported that field populations of WCR resistant to Cry3Bb1 were cross resistant to other Cry3-based traits, mCry3A and eCry3.1Ab (5, 6). In 2016 and 2017, field populations of WCR resistant to Cry34Ab1/Cry35Ab1-traited maize also were reported (7, 8). In 2020, field populations of WCR resistant to maize expressing both Cry3Bb1 and Cry34Ab1/Cry35Ab1 were reported (9). The discovery of these field populations highlights the need to bring to the market new molecules to control WCR in maize that are not cross resistant to Cry3Bb1 and Cry34Ab1/Cry35Ab1.

Since the late 1990s, the investigation of insecticidal bacteria has intensified and expanded beyond B. thuringiensis to other endospore-forming bacilli, including Lysinibacillus sphaericus (10–12) and Brevibacillus laterosporus (13). Further, non-spore-forming common soil bacteria like *Chromobacterium* subspecies and *Pseudomonas* subspecies have also generated considerable interest and research. The most promising results reported for control of WCR are a novel protein from *Pseudomonas* spp. (14, 15) and from Chromobacterium piscinae (16), both of which have been shown to reduce WCR root damage in GM maize.

Like B. thuringiensis, B. laterosporus is a member of the *Firmicutes* division of bacteria and are Gram-positive endospore-forming bacilli. Formerly known as Bacillus laterosporus, B. laterosporus was isolated originally from water and characterized in 1916 (17). While the bacterium was assigned a new genus in 1996 to distinguish *Brevibacillus* from other members of the genus *Bacillus* (18), it is still a firmicute and shares many similarities to other endospore-forming insecticidal bacilli, including B. thuringiensis. B. laterosporus is ubiquitous and has been isolated from different environmental sources around the world and from a wide range of materials, including various soil samples, fresh- and seawater, insects and other animals, leaf surfaces, and many types of food material (19, 20). B. laterosporus strains have been shown to be insecticidal against some Diptera, Lepidoptera, and Coleoptera insect pests (19, 21, 22), and in the only two reports that evaluated B. laterosporus against insects outside these three orders, Muscidifurax raptor (Hymenoptera), and Chrysoperla agilis (Neuroptera) (23, 24), no or only slight toxicity was observed. Most of the studies on insecticidal spectrum or host range have relied on testing crude fractions or preparations containing viable B. laterosporus cells, making it difficult to understand specific components or factors contributing to toxicity and virulence. A recent study describes the phylogenetic analysis and toxin gene distribution in genome sequences of B. laterosporus strains (25). Additionally, a commercial B. laterosporus-based product, Lateral, will be released in New Zealand during the 2020 season pending Environmental Protection Authority (EPA) and Agricultural Compounds and Veterinary Medicines (ACVM) consents (https://www.hortibiz.com/news/?tx_news_pi1%5Bnews%5D=31932&cHash=a202ca0db6f60c60552da81b03cd7da1). B. laterosporus also is commercially available as a human probiotic supplement, known by the commercial name Latero-Flora.

This study describes the isolation and characterization of three new strains of endospore-forming bacteria putatively identified as B. laterosporus. Several matches to known insecticidal proteins with low sequence identity to known insecticidal proteins were identified from the whole-genome draft sequence of each of the three strains. Three proteins identified in the whole-genome draft sequences, based on Pfam hits to ETX_MTX2 (26), were cloned and expressed in bacteria. These proteins were fed to a panel of coleopteran pests, and insecticidal activity was only observed with WCR. The three protein sequences were submitted to the Bacillus thuringiensis nomenclature committee of 2017 and were assigned the designations Cry75Aa1, Cry75Aa2, and Cry75Aa3. Recently, the nomenclature was revised (https://www.bpprc.org/), clustering proteins based on homologous structure with the understanding that proteins sharing sequence homology are likely to equally share structural configurations. As a result, the Cry75Aa proteins have been assigned the new designations of Mpp75Aa1, Mpp75Aa2, and Mpp75Aa3 (2, 27; https://www.bpprc.org/). Transgenic plants expressing each mMpp75Aa protein were protected from feeding damage and showed a significant reduction in adult emergence from infested plants by both susceptible and Cry3Bb1 and Cry34Ab1/Cry35Ab1-resistant WCR. These results demonstrate that proteins from B. laterosporus are as efficacious as the well-known B. thuringiensis insecticidal proteins at controlling major insect pests such as WCR.

## RESULTS

### Isolation and genomic characterization of the Mpp75Aa source strains.

**FIG 1:**
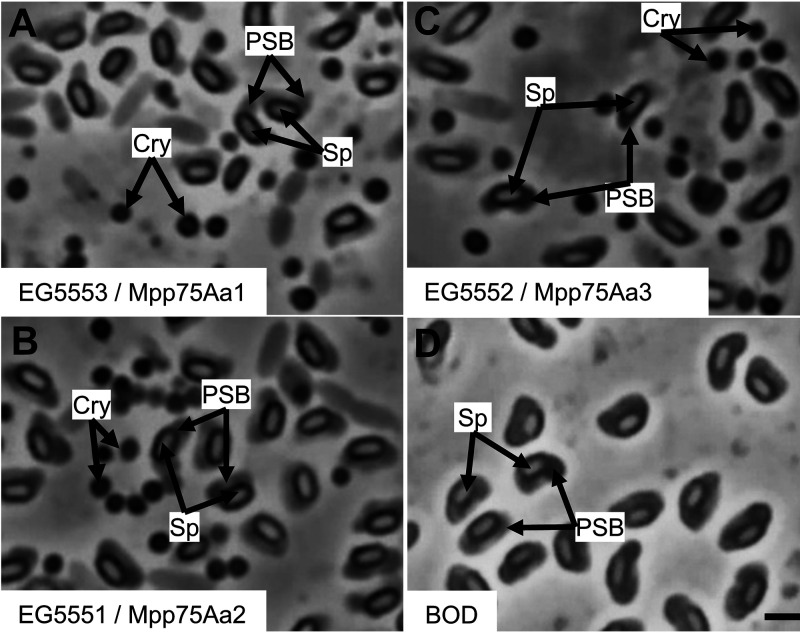
Phase contrast micrographs of the Mpp75Aa containing wild-type B. laterosporus strains. All the strains show the typical morphology of the canoe-shaped parasporal-body (CSPB) alongside the spore (Sp). The EG strain also show the presence of crystal proteins (Cry). (A) EG5553 source of Mpp75Aa1. (B) EG5551 source of Mpp75Aa2. (C) EG5552 source of Mpp75Aa3. (D) B.O.D., human probiotic strain.

**FIG 2:**
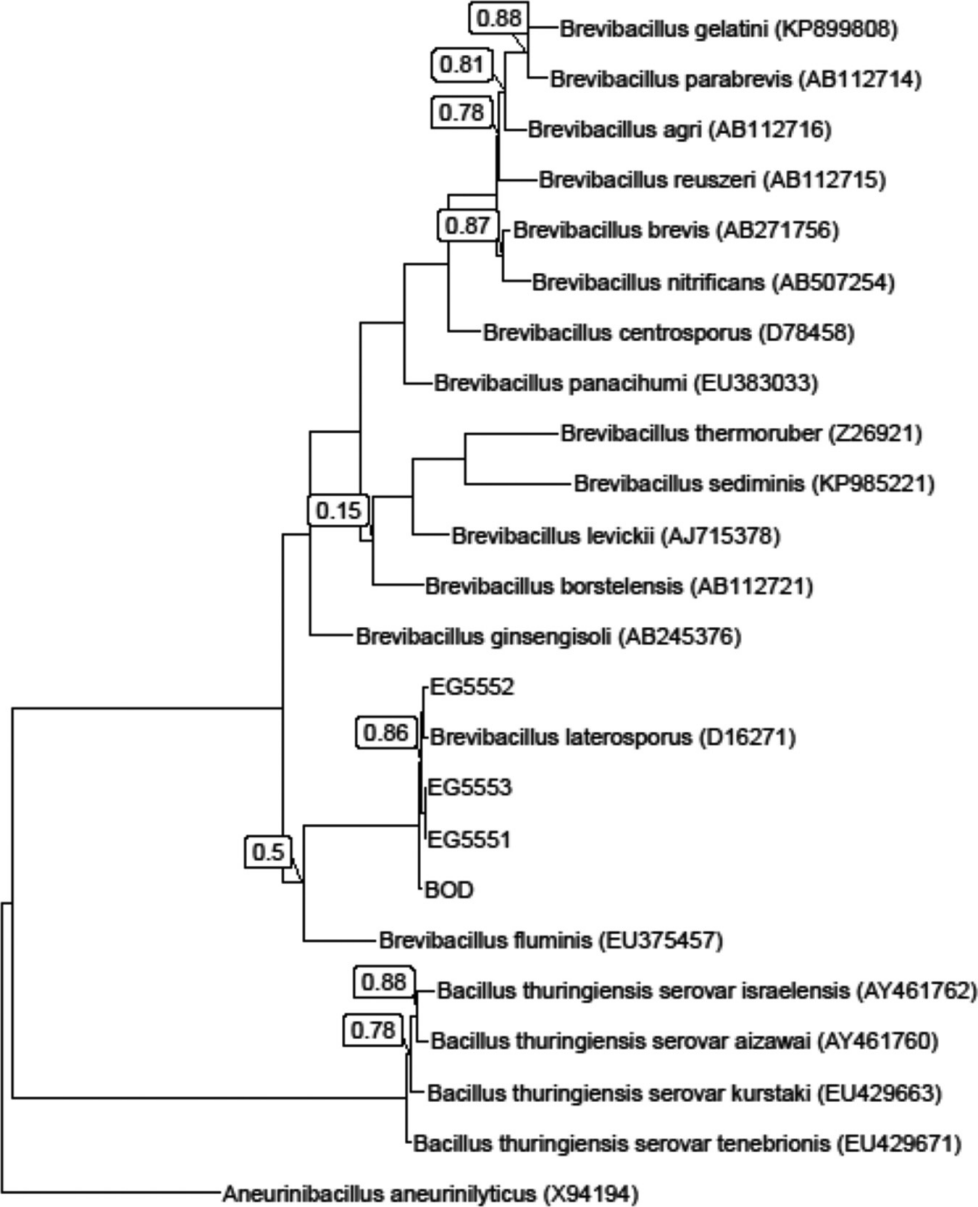
Brevibacillus laterosporus strains form a well-supported clade in the genus *Brevibacillus*. A 16S rRNA gene maximum-likelihood phylogeny is shown, containing gene sequences from strains of select species in the genera *Brevibacillus* and *Bacillus*, with Aneurinibacillus aneurinilyticus included as an outgroup. Gene sequences obtained from the public have an accession number specified in parentheses, and entries without parentheses are sequenced reported in this work. Internal nodes have a bootstrap support value of >0.9, except where that value is shown.

The three source strains for Mpp75Aa from B. laterosporus were isolated from grain dust, and the B.O.D. strain was isolated from the powder of a commercially available probiotic capsule. Cells picked from well-isolated colonies were examined by phase-contrast microscopy (Fig. 1). The bacteria were presumptively identified as B. laterosporus based on the presence of a spore (a crystal protein in the EG strains) and the presence of the typical canoe-shaped parasporal body (CSPB) firmly attached to one side of the spore (19, 28). Further taxonomic classification was achieved through whole-genome sequencing and analysis of the 16S rRNA. Genomic DNA was subjected to massively parallel sequencing on the Illumina HighSeq platform to ∼100× coverage based on an estimated 5.5-Mb genome size. A draft-quality assembly was created using standard annotation methods that predicted and identified genes. The 16S rRNA gene sequence for each of the strains was identified in the genome assembly, and these sequences were used to construct a 16S rRNA gene maximum-likelihood phylogeny comparing the four strains in this study with publicly available *Brevibacillus* sp., B. thuringiensis, and other bacilli (Fig. 2). The four strains form a well-supported clade with the type strain B. laterosporus IAM 12465 (NCBI nucleotide accession no. D16271) in the genus *Brevibacillus*.

Genes from the three EG strains were translated into protein sequences and subjected to further analysis, including BLAST against public protein databases, Ffam analysis, and a custom protein database of insecticidal protein curated and maintained at Bayer Crop Science. Results of these analyses demonstrated that each of the EG strains carried several known insecticidal proteins in their genomes. All three EG strains had a Cry8Ad homolog and two different Vpb1/Vpa2 operons, with one encoding Vpb1Ba/Vpa2Ba and the second encoding Vpb1Da/Vpa2Ad (Table 1). Each EG strain was found to contain a unique protein, identified by Pfam analysis as belonging to the ETX_MTX2 family, and each sharing low sequence identity to known insecticidal proteins. These proteins were designated TIC3668, TIC3669, and TIC3670. A PCR product generated from the genomic DNA of the three EG strains was cloned into an E. coli/B. thuringiensis shuttle vector. The gene of interest (GOI) was sequenced from the isolated plasmid DNA from E. coli, and the sequence for each of the three GOIs matched the sequence draft genome sequence. Each gene is 884 nucleotides in length (including a stop codon), resulting in a full-length protein of 317 amino acids. The SignalP algorithm predicted the first 23 to be a membrane-transiting signal peptide that would be cleaved between amino acids 23 and 24, as the protein is secreted from the host cell and most likely confers no effect on insecticidal activity. The three protein sequences were submitted to the Bacillus thuringiensis nomenclature committee and received official Cry designations of Cry75Aa1 for TIC3670, Cry75Aa2 for TIC3669, and Cry75Aa3 for TIC3668 (http://www.lifesci.sussex.ac.uk/home/Neil_Crickmore/Bt/). In July 2020, the existing nomenclature was revised, and, as a result, these proteins were designated Mpp75Aa1, Mpp75Aa2, and Mpp75Aa3 (https://www.bpprc.org) (2).

**TABLE 1:** Known insecticidal proteins in the genome sequence of the Brevibacillus laterosporus strains in this study^*a*^

Strain	Mpp75Aa protein	Cry8Ad homolog^*b*^	VIP1/VIP2 operons
EG5553	Mpp75Aa1	Cry8Ad (100% ID)	Vpb1Ba/Vpa2Ba; Vpb1Da/Vpa2Ad
EG5551	Mpp75Aa2	Cry8Ad (100% ID)	Vpb1Ba/Vpa2Ba; Vpb1Da/Vpa2Ad
EG5552	Mpp75Aa3	Cry8Ad (4 aa difference)	Vpb1Ba/Vpa2Ba; Vpb1Da/Vpa2Ad

a The draft genome assembly predicts the presence of single copies of Mpp75Aa in each EG strain. Also identified in the genome assembly within each strain were the genes encoding Cry8Ad and encoding two operons for Vpb1/Vpa2 proteins.

b ID, identity.

**FIG 3:**
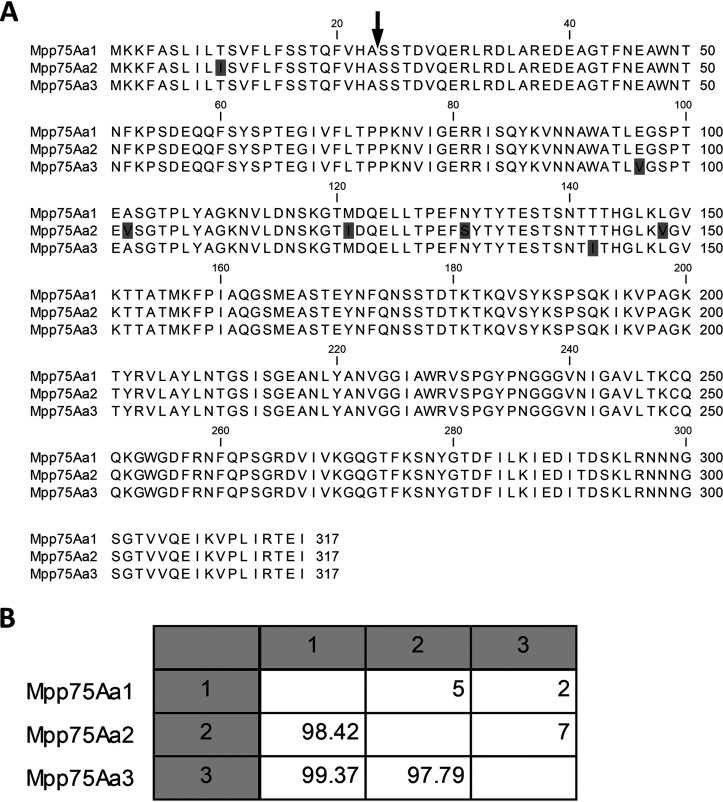
Multiple-sequence alignment and pairwise comparison of the amino acid sequence of Mpp75Aa. (A) Multiple sequence alignment of Mpp75Aa homologs was built using ClustalW with the full-length amino acid sequences. The arrow between amino acids 23 and 24 shows the site where the N-terminal membrane-transiting signal peptide is cut and removed and the start of the mature form of the protein. The shaded boxes are amino acids that differ from the consensus sequence. (B) Pairwise comparison of the Cry75Aa (Mpp75Aa) homologs. The numbers in the lower left boxes are the percent identity between the sequences, and those in the upper right are the number of amino acid differences between the pairs.

The three protein sequences were aligned to each other in a multiple-sequence alignment (Fig. 3A) followed by a pairwise comparison (Fig. 3B). The three Mpp75Aas were all >97% identical to each other, with a small number of amino acid differences occurring in the first half of the proteins.

### Expression of Mpp75Aa genes and insecticidal activity.

The three Mpp75Aa proteins produced in either the E. coli or B. thuringiensis expression systems yielded sufficient protein quantities for insect diet bioassays. Insect diet was overlaid with buffer solutions containing 15.2 µg/cm^2^ for mMpp75Aa1 and mMpp75Aa3 and 43.4 µg/cm^2^ for mMpp75Aa3 that were produced in E. coli. The three mCry75Aa proteins were tested on WCR, southern corn rootworm (SCR), and Colorado potato beetle (CPB), resulting in high levels of mortality and stunting on WCR but no activity on SCR and CPB (Table 2). The mMpp75Aa homologs expressed in E. coli or Mpp75Aa expressed in an atoxigenic B. thuringiensis host as an insoluble CSP, or the solubilized fraction of a CSP had similar levels of insecticidal activity on WCR (our unpublished results).

**TABLE 2:** Coleopteran stunting and mortality when fed mMpp75Aa^*a*^

Cry type	Concn (µg/cm^2^)	Activity of:		
		WCR	SCR	CPB
mMpp75Aa1	15.2	+++	—	—
mMpp75Aa2	43.4	+++	—	—
mMpp75Aa3	15.2	+++	—	—

a Each of the mCry75Aa (mMpp75Aa) proteins caused severe stunting and mortality when fed to WCR in diet bioassay overlay. No effect was observed when fed to SCR or CPB. —, no activity; +, low stunting where the insect larvae are 50 to 90% the size of larvae fed the buffer control; ++, moderate stunting and mortality where the larvae are 25 to 50% the size of larvae fed the buffer control and/or less than 50% mortality; +++, severe stunting and mortality where the larvae are less than 25% the size of larvae fed the buffer control and/or greater than 50% mortality.

### mMpp75Aa homologs in transgenic maize evaluated in growth chamber testing.

Plant transformation and expression constructs were built with the three Mpp75Aa genes encoding the full-length protein, the mature form (mMpp75Aa), or the mMpp75Aa with a chloroplast-targeting peptide (CTP) encoded at the 5′ end of the gene in a translational fusion. Constructs designed to express the full-length Mpp75Aa protein were transformed into maize. F1 single-copy transformed plants were identified and tested in growth chamber pot assays for the ability to protect root from feeding by susceptible WCR.

**FIG 4:**
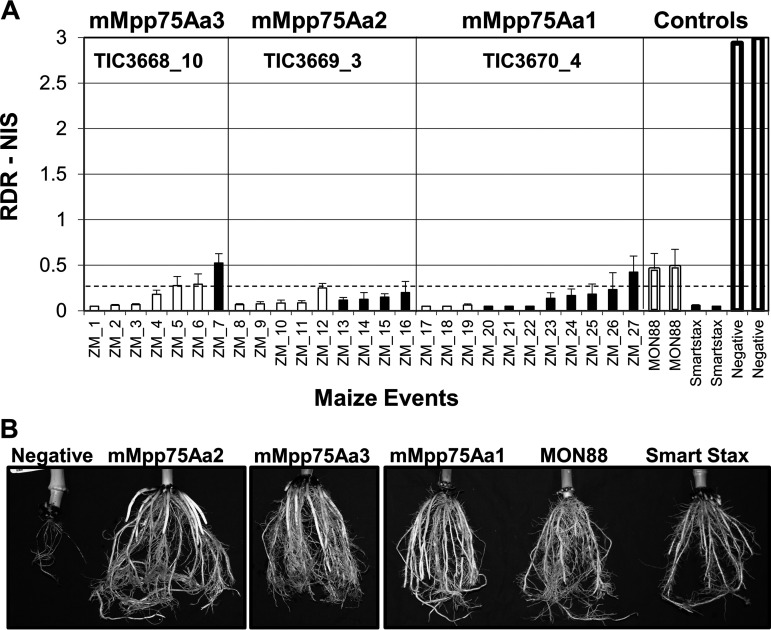
Growth chamber testing of WCR on transgenic maize. Evaluation of root protection from feeding damage by WCR on transgenic maize expressing mMpp75Aa homologs. (A) Root damage rating of node injury scores (RDR-NIS) of F1 maize infested at the V4 stage with 2,000 WCR eggs and roots rated 24 days postinfestation compared with 3 different maize controls, including (i) transgenic MON88 expressing Cry3Bb1, (ii) SmartStax expressing Cry3Bb1 and Cry34Ab1/Cry35Ab1, and (iii) the negative nontransgenic isoline maize. The dashed line represents the level of root protection for a commercial product. The black bars are events expressing mMpp75Aa1 with an N-terminal CTP. (B) Photographs of representative plants from each group showing normal root architecture with minimal feeding damage by WCR on the transgenic mMpp75Aa or the transgenic positive controls. The dashed line represents the level root protection for a commercial product. The error bars show the standard error of the mean (SEM).

**FIG 5:**
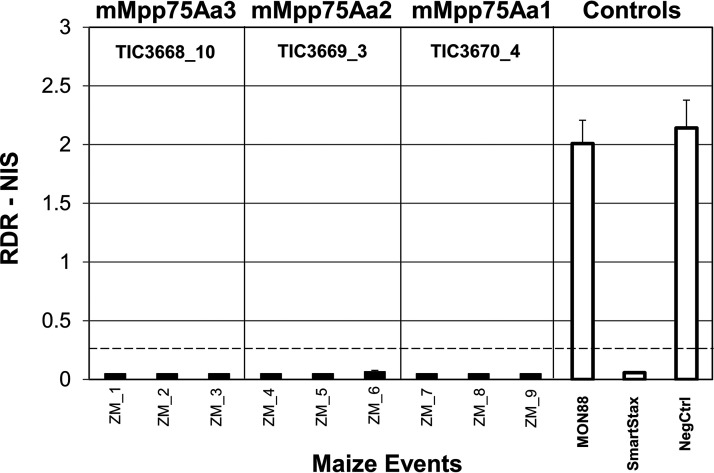
Growth chamber testing of Cry3Bb1-resistant WCR on transgenic maize. Evaluation of root protection from feeding damage by Cry3Bb1-resistant WCR on F1 generation transgenic maize expressing the mature form of the mMpp75Aa homologs. Root damage rating of node injury scores (RDR-NIS) of F1 maize infested at the V4 stage with 2,000 Cry3Bb1-resistant WCR eggs (from a field-derived Cry3Bb-resistant strain) and roots rated 24 days infestation compared with 3 different control maize, transgenic MON88 expressing Cry3Bb, SmartStax commercial line expressing Cry3Bb1 and Cry34Ab1/Cry35Ab1, and the negative control nontransgenic isoline maize. The dashed line represents the level root protection for a commercial product with an NIS score of <0.25. The error bars show the SEM.

The F1 plants expressing the full-length mMpp75Aa homologs failed to protect the roots from feeding damage caused by the WCR larvae. All events for the three mMpp75Aa homologs with or without a CTP showed root protection from feeding damage by WCR, and 25 of 27 events had commercial-level root protection defined as a node injury scale (NIS) score of <0.25 (Fig. 4A). All plants tested were stable, single-copy, transgenic events expressing ∼4 to 8 ppm (dry weight) in root tissue (our unpublished results). No abnormal phenotypes were observed in any transgenic maize expressing mMpp75Aa homologs, and normal root architecture with minimal feeding damage by WCR was observed (Fig. 4B). Commercial-level root protection from Cry3Bb1-resistant WCR feeding damage was also observed with plants expressing the three mMpp75Aa homologs (Fig. 5).

### mMpp75Aa1 transgenic maize evaluated in field testing.

**FIG 6:**
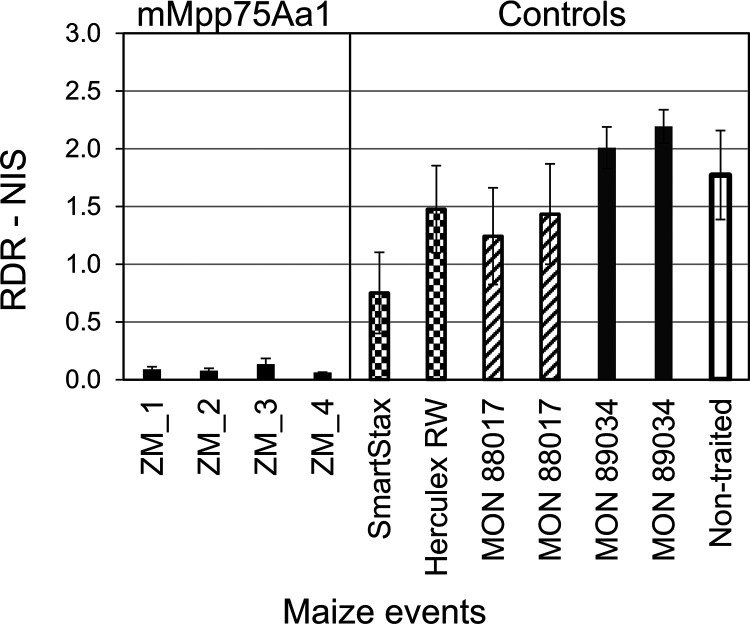
mMpp75Aa1 transgenic maize lines demonstrate commercial-level efficacy of root protection in fields with high WCR pressure and resistance to Cry3Bb1 and Cry34Ab1/Cry35Ab1. Levels of root damage rated in node injury score (NIS) (50) are shown from 2017 field trial at locations with high WCR pressure (Colesburg, Iowa, and Fairbank, Iowa). The four mMpp75Aa1-expressing maize lines showed excellent root protection, while the current commercial traits MON88017 expressing Cry3Bb1, Herculex RW expressing Cry34Ab1/Cry35Ab1, SmartStax expressing both Cry3Bb1 and Cry34Ab1/Cry35Ab1, the Lepidopteran protection-only trait MON89034, and nontraited isoline showed moderate to severe root feeding damage. The error bars show the SEM.

Transgenic maize lines expressing mMpp75Aa1 were further evaluated in field locations where greater than expected damage (GTED) by WCR was detected in the previous season (29). At locations exhibiting the highest WCR pressure in early season evaluations, severe larval root damage with an NIS score of ∼2.0 was observed on maize lines carrying either no insect protection traits or the Lepidopteran protection trait MON89034 (Fig. 6). Substantial root damage (NIS score, 0.75 to 1.50) was observed on commercially available corn rootworm protection traits, including MON88017 expressing Cry3Bb1 in VT Triple Pro, DAS-59122-7 expressing Cry34Ab1/Cry35Ab1 in Herculex RW, and the pyramided MON88017 × DAS-59122-7 in SmartStax. In contrast, transgenic maize lines expressing mMpp75Aa1 were well protected, exhibiting NIS scores of <0.13, indicating that mMpp75Aa1 expressed in maize can deliver commercial-level field protection (NIS score <0.25) against high-pressure WCR field populations likely containing resistance to Cry3Bb1 and Cry34Ab1/Cry35Ab1.

Maize lines expressing different levels of mMpp75Aa1 were also tested for their ability to reduce adult beetle emergence in two fields with historically high WCR damage and suspected resistance to Cry3Bb1 and Cry34Ab1/Cry35Ab1 (Table 3). High numbers (>49 adults per plant) of adult WCR emerged in tents planted with maize carrying no corn rootworm protection trait, indicating high WCR pressure at the two locations. Relatively few WCR adults were recovered from tents planted with mMpp75Aa1 transgenic maize, resulting in a percent reduction in adult emergence of 98.8% (low expression) to 99.8% (high expression) (Table 3).

**TABLE 3:** Adult emergence from mMpp75Aa1 lines is dramatically reduced at GTED sites^*a*^

Maize line	Site	Total no. of WCR beetles	No. of WCR beetles/plant	Reduction in beetle emergence (%)
mMpp75Aa1^*b*^	Leigh	218	0.61	98.8
	Shelby	123	0.37	99.5
mMpp75Aa1^*c*^	Leigh	134	0.38	99.2
	Shelby	50	0.15	99.8
Nontraited	Leigh	17,660	49.06	
	Shelby	24,915	74.15	

a Six replications of isoline- and mMpp75Aa1-expressing maize were planted in Nebraska at two locations (Leigh and Shelby) with putative high WCR pressure in late April to early May 2017. Normal agronomic practices for the area were followed, and no insecticides were applied at any time. Both low and high Mpp75Ab1-expressing maize showed dramatic reductions in beetle emergence.

b Low expression.

c High expression.

### mMpp75Aa1 is not cross resistant with currently registered and next-generation WCR control traits.

To further investigate field observations that mMpp75Aa1 provided protection against WCR populations causing damage to Cry 3Bb1 and Cry34Ab1/Cry35Ab1 maize, a series of studies were conducted to evaluate mMpp75Aa1 against WCR colonies resistant to Cry3Bb1 (30), Cry34Ab1/Cry35Ab1 (31), and DvSnf7 double-stranded RNA (dsRNA) that is expressed in the recent U.S. Environmental Protection Agency-registered SmartStax Pro (32).

**FIG 7:**
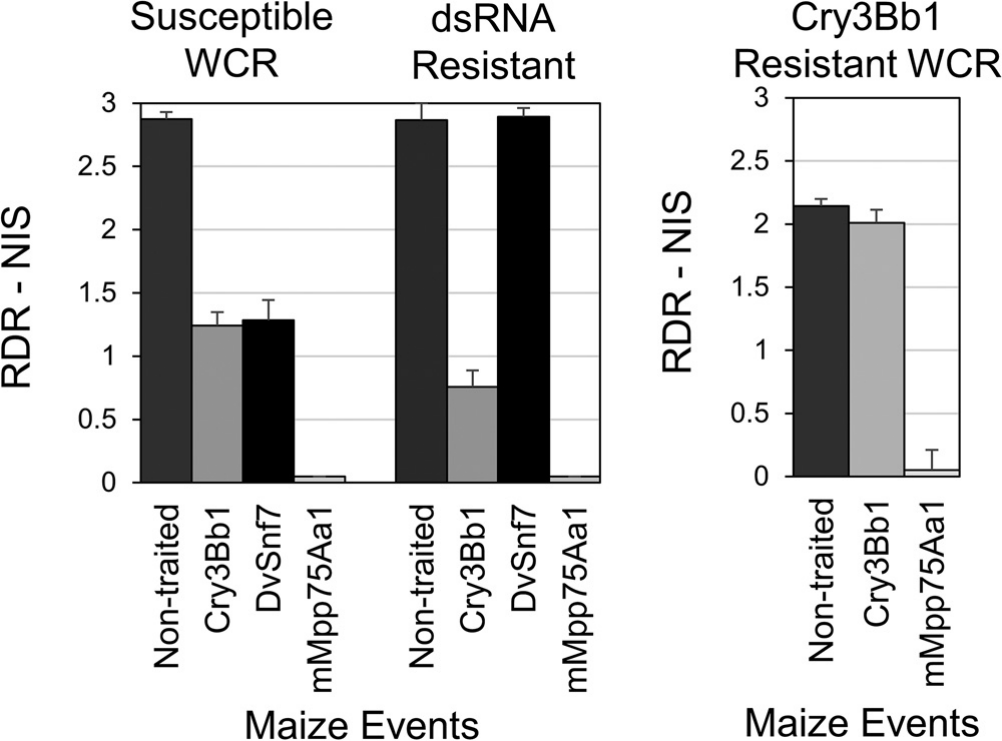
mMpp75Aa1 is not cross resistant with Cry3Bb1 or DvSnf7 RNA against WCR. RDR-NIS are shown from growth chamber root protection assays, in which 2,000 eggs from susceptible (Waterman, Illinois), Cry3Bb1-resistant WCR, or dsRNA-resistant WCR colonies were infested on a single maize plant from either nontransgenic isoline or transgenic lines expressing Cry3Bb1 or DvSnf7 RNA. The error bars show the SEM.

In preliminary diet-overlay bioassays, similar levels of mortality were observed when mMpp75Aa1 was fed to susceptible or Cry3Bb1-resistant WCR neonates (our unpublished results). In growth chamber root protection assays, nontransgenic maize sustained significant root damage (NIS score >2.5) by all WCR colonies evaluated (Fig. 7). The susceptible colony produced moderate damage on the Cry3Bb1 and DvSnf7 dsRNA-expressing maize lines (NIS score of 1.2 to 1.3) but caused negligible damage (NIS score, ∼0.1) to the mMpp75Aa1-expressing maize lines. Cry3Bb1-resistant WCR larvae caused high damage to both the nontraited and the Cry3Bb1-expressing maize as expected and a moderate level of damage (NIS score, ∼1.3), on the DvSnf7 transgenic line, but they caused essentially no damage on the Mpp75Aa1-expressing maize. The DvSnf7 dsRNA-resistant WCR caused similar high damage to the DvSnf7 dsRNA-expressing line compared to the nontransgenic maize, as well as moderate damage (NIS score, ∼0.7) on the Cry3Bb transgenic line. Negligible damage was observed when infested on mMpp75Aa1-expressing maize (Fig. 7). These data demonstrate that mMpp75Aa1 is not cross resistant with Cry3Bb1 or DvSnf7 dsRNA.

**FIG 8:**
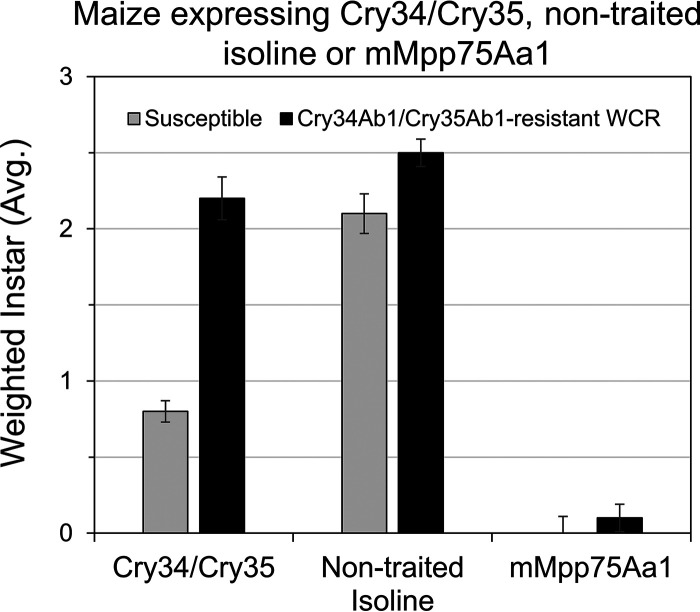
There is no cross-resistance between Cry34Ab1/Cry35Ab1-resistant WCR and mMpp75Aa1. The left gray bar and right gray bar confirm the sensitivity with no later-stage (2nd to 3rd instars)-susceptible larvae recovered after feeding on Cry34/Cry35 or mMpp75Aa1 maize. Both the center gray and black bars confirm from the nontraited isoline maize with only late-instar (2nd to 3rd) larvae were recovered on both susceptible and Cry34Ab1/Cry35Ab1-resistant WCR. The left black bar confirms the resistance of the Cry34Ab1/Cry35Ab1-resistant WCR larvae with only later-instar (2nd to 3rd) larvae recovered on Cry34Ab1/Cry35Ab1, and the right black bar shows no later instars (2nd to 3rd) recovered on mMpp75Aa1 maize. The error bars show the SEM.

In 10-day larval recovery assays implemented to evaluate the potential for cross-resistance with Cry34Ab1/Cry35Ab1 using a field-originated Cry34Ab1/Cry35Ab1-resistant WCR colony, WCR larval development was quantified using the larval instar score (LIS) in the range of 0 to 3. Both the Cry34Ab1/Cry35Ab1-resistant WCR and a susceptible WCR colony were recovered with an LIS of 2.1 to 2.5 when feeding on the nontraited maize isoline, indicating the expected normal growth rate in the test (Fig. 8). A slightly yet statistically significant higher LIS for the resistant colony suggests slightly higher fitness in this population. When feeding on the Cry34Ab1/Cry35Ab1-expressing Herculex RW, a significant increase in mortality and delay in development was observed for the WCR control-susceptible colony (LIS, ∼0.8; a value of <1 indicates either early mortality or molting not yet reaching 2nd instar) but not for the Cry34Ab1/Cry35Ab1-resistant WCR (LIS, ∼2.2), confirming that this resistant colony is indeed resistant to Cry34Ab1/Cry35Ab1. In contrast, feeding on maize plants expressing mMpp75Aa1 caused significant mortality and developmental delay (LIS, 0.1) for the Cry34Ab1/Cry35Ab1-resistant WCR. In addition, feeding on maize plants expressing mMpp75Aa1 caused the lowest LIS (0.0 to 0.1) in the test for both the Cry34Ab1/Cry35Ab1-resistant WCR and susceptible WCR, suggesting that this mCry75Aa1 transgenic line is more toxic than the Herculex RW against WCR. This study confirms the field observation of lack of cross-resistance between mMpp75Aa1 and Cry34Ab1/Cry35Ab1.

## DISCUSSION

The WCR is the most damaging maize pest in North America and has been nicknamed the “billion-dollar bug” because the yield losses and cost of control measures annually, in the United States alone, exceed a billion dollars. Resistance to chemical insecticides has readily emerged in WCR populations and spread across maize-growing regions. The most successful strategy for protection of maize from feeding damaging by WCR has been the deployment of GM maize in the United States.

This study identified three new proteins from the bacterium Brevibacillus laterosporus (formerly known as Bacillus laterosporus) (18) with insecticidal activity on WCR. Through whole-genome sequencing of endospore-forming crystal-forming bacilli, we identified potential insecticidal proteins using bioinformatic analysis of the genome sequence. The first classification of these isolates as B. laterosporus was by observing the distinct fusiform morphology of the canoe-shaped parasporal body (CSPB) first described in 1916 (17). Confirmation of the morphological identification by analysis of 16s rRNA sequence from the genome sequence of our strains further established the three new WCR active proteins were from B. laterosporus.

The genome sequence data produced from this study showed the B. laterosporus strains EG5551, EG5552, and EG5553 carried several well-known insecticidal proteins, Cry8 and Vpb1s/Vpa2s, in addition to the novel 3 Mpp75Aa homologs, which are members of the MTX2 family of proteins. This is further evidence of the diversity of the toxin profiles among B. laterosporus strains (25). The insect diet bioassay testing of the three mMpp75Aa proteins showed they were insecticidal when fed to WCR but had no insecticidal activity when fed to two other coleopteran species, SCR and CPB (Table 2). The MTX2 family of proteins includes ∼40 known insecticidal proteins which have been assigned the Mpp family designation and show insecticidal activity on a variety of insects.

The most significant results of this study came when the mMpp75Aa proteins were expressed in maize. All three proteins expressed in their mature form separately in maize provided protection from root feeding damage by WCR in growth chamber and field experiments. Interestingly, expression of full-length proteins (Mpp75Aa) resulted in no WCR control when expressed in maize, although they were toxic to coleopterans when expressed in bacteria. This is probably due to the inability of plants to read bacterial signal peptides either blocking protein translation or inhibiting protein secretion into the plant cytoplasm.

In the first set of experiments using lab-susceptible WCR larvae, GM maize events expressing provided commercial-level root protection from feeding damage, lacked adverse phenotypes, and showed normal root architecture. All three mMpp75Aa proteins were highly efficacious in reducing damage by WCR, which should provide insight into regions of these proteins most responsible for WCR activity. In further GM maize testing in the growth chamber using WCR resistant to current registered B. thuringiensis products, plants expressing mMpp75Aa provided root protection from feeding damage by WCR resistant to Cry3Bb1, Cry34Ab1/Cry35Ab1, and DVSnf7-dsRNA. Although all three mMpp75Aa proteins were toxic to WCR in field efficacy studies, mMppAa1 was chosen for additional studies because of its high level of efficacy across most events. Field trial experiments at GTED locations evaluating damage and adult emergence (the most common method to evaluate mortality and dose of a WCR-traited plant) against WCR, most likely resistant to either Cry3Bb1, Cry34Ab1/Cry35Ab1, or both, also resulted in a commercially acceptable control. Although simultaneous tests would need to be performed to confirm it, in data in published reports on next-generation WCR traits that have been submitted for regulatory approval, mMpp75Aa1 appears to provide a comparable or greater level of root protection than DvSnf7 RNA (32) or the Pseudomonas chlororaphis protein, IPD072Aa (14).

There is a continuing need to discover and evaluate insecticidal molecules from a variety of known and newly described bacterial sources to provide alternative solutions capable of controlling resistant populations of insect pests. Besides proteins isolated from Pseudomonas chlororaphis (14) and Chromobacterium piscinae (16), this study has demonstrated that Mpp75Aa proteins from B. laterosporus are as efficacious as the well-known B. thuringiensis insecticidal proteins at controlling WCR in both insect diet bioassay experiments as well as in transgenic maize expressing these proteins. Additionally, multiple experiments using transgenic maize expressing mMpp75Aa proteins have demonstrated commercial-level root protection from feeding damage by susceptible WCR and B. thuringiensis-resistant WCR.

## MATERIALS AND METHODS

### Isolation and growth of B. laterosporus strains.

B. laterosporus strains EG5553, EG5551, and EG5552 were sourced from grain dust samples obtained from farmers in the eastern and central regions of the United States, and the strains described in this study were isolated in 1986. Grain dust samples were treated as described in Donovan et al. (33, 34) and spread on NYSM agar plates to isolate *Bacillus*-type colonies. Phase-contrast microscopy of sporulating colonies identified B. laterosporus-type sporangia production with the typical canoe-shaped parasporal body (CSPB) firmly attached to one side of the spore. Isolated B. laterosporus strains were further analyzed by a modified Eckhardt agarose gel electrophoresis procedure (35) to determine the array of native plasmids found in each strain. Strains with unique plasmid arrays and crystal morphologies were selected for further study. The B.O.D. strain was isolated from capsule powder of the commercially available Latero-Flora human dietary supplement. The powder was resuspended in 1 ml of sterile water, heat shocked in an 80°C water bath for 20 min, plated on NYSM agar, and incubated at 28°C for 3 days. Colonies were picked and examined under phase-contrast microscopy to identify B. laterosporus-type sporangia with the typical CSPB firmly attached to one side of the spore (19, 28).

### DNA isolation.

Cultures of EG5551, EG5552, EG5553, and B.O.D. for DNA extraction were initiated from −80°C glycerol stocks. Each culture was started by adding 100 µl to 900 µl of sterile terrific broth (TB) to a deep-well 96-well plate, covered with an AirPore sheet, and placed in a Multitron shaker at 28°C with shaking at 900 rpm overnight. The next day, 2 wells containing fresh 900 µl TB were inoculated with 100 µl of the overnight culture, covered with an AirPore membrane, and placed back on the Multitron at 28°C and 900 rpm for ∼3 to 4 h. Plates were centrifuged at 2,700 × *g* for 10 min in an S5700 swinging bucket rotor (Beckman Coulter), the supernatant was discarded, and pellets were washed with 900 µl/well of sterile 1 M NaCl and centrifuged again at 2,700 × *g* for 10 min and placed at −20°C until DNA isolation.

Qiagen DNeasy blood and tissue DNA kits were used for DNA isolation with several modifications to the protocol described below (Qiagen, Germantown, MD). Bacterial cell pellets were thawed at room temperature for 30 min, and 400 µl of a lysis buffer cocktail consisting of 10 mM Tris-HCl, pH 8.0, 0.5 mM EDTA, pH 8.0, 0.005% Triton X-100, 0.5% Tween 20, 2 µl RNase A (20 mg/ml), and 2 µl Riboshredder was added, fully resuspending bacterial cell pellets. Using a heated shaker platform, samples were incubated at room temperature for 10 min (no shaking), 37°C for 10 min (with slow shaking), and 56°C for 30 min (with slow shaking). Then 200 µl AL buffer (no ethanol added) and 25 µl of proteinase K µg/ml were added to each well. Samples were incubated at 65°C for 10 min with slow shaking, and finally, 350 µl of ethanol was added. From each sample, 900 µl of lysate was added to the kit purification plate. The plate was sealed and centrifuged at 4,300 × *g* for 30 min. Then, 500 µl/well of AW1 buffer was added to the plate, sealed, and centrifuged for 30 min at 4,300 × *g*. We then added 500 µl/well of AW2 buffer, and the plate was centrifuged at 43,00 × *g* for 15 min. The column plate was placed over the elution plate, 160 µl/well of AE elution buffer was added and let sit for 1 min, and then the plate was sealed and centrifuged at 2,700 × *g* for 10 min. DNA samples were quantified using a Trinean 96-well DNA microfluidics plate reader. To each lane, 2 µl of DNA was added and analyzed for DNA concentration and purity.

### Genome sequencing.

Total extracted genomic DNA from, EG5553, EG5551, EG5552, and B.O.D. was subjected to massively parallel sequencing on the Illumina HighSeq platform using the 2 × 100-bp paired-end format. DNA sequencing libraries were prepared using the Nextera library prep method (Illumina Inc., San Diego, CA). Reads were assembled using the CLCBio Genomic Workbench or the SPAdes algorithm. Assembled contigs were analyzed using a custom bioinformatic pipeline developed by Bayer Crop Science for gene calling and protein annotation. The bioinformatic analysis included BLAST against public protein databases (Swiss-Prot and Uniref100), Pfam analysis, SignalP, which predicts the presence of membrane-transiting signal peptides and the location of their cleavage (36, 37), and a custom insecticidal protein database curated and maintained at Bayer Crop Science.

### Phylogeny construction by 16S rRNA analysis.

A phylogeny of select endospore-forming bacilli was constructed using the ribosomal 16S RNA sequence (16S rRNA) (38). The 16S rRNA gene sequences were obtained from NCBI or generated from genome sequences of isolates described in this study with barrnap version 0.8 (https://github.com/tseemann/barrnap). Gene sequences were aligned using cmalign (INFERNAL 1.1.3 suite) (39) using the Rfam model for the small subunit rRNA to guide alignment (RF00177; http://rfam.xfam.org/family/RF00177). The resulting alignment was used to infer a maximum-likelihood phylogeny with FastTree version 2.1.10 SSE3, using the default options (40), and the resulting tree was visualized with ggtree (41).

### Mpp75Aa identification, cloning, and expression.

The full-length genes encoding TIC3670, TIC3669, and TIC3668 were putatively identified in the draft genome assemblies based on Pfam analysis and BLAST hits to known insecticidal proteins. PCR primers were designed based on the first 30 nucleotides of the 5′ end and the last 30 nucleotides of the 3′ ends of the coding region to generate PCR products. An additional 16 nucleotides were added to both 5′ and 3′ ends to enable hot fusion cloning (42). PCR products were generated using standard PCR conditions with KOD hot start DNA polymerase (Novagen, San Diego, CA). The same DNAs used for the whole-genome sequencing were used to generate PCR products for hot fusion. PCR products were cloned into a B. thuringiensis expression/E. coli shuttle vector (designed and created at Bayer Crop Science). The mature forms of the three proteins (mMpp75Aa; membrane-transiting signal peptide removed) were subcloned into E. coli strain DH10B. Plasmid DNA from E. coli clones containing TIC3668, TIC3669, and TIC3670 (with/without signal peptides) was isolated and sequence confirmed by Sanger sequencing (ABI 3700). The three confirmed protein sequences were submitted to the Bacillus thuringiensis Nomenclature Committee and assigned Cry designations (http://www.lifesci.sussex.ac.uk/home/Neil_Crickmore/Bt/). Recently, the BPPRC reviewed the three proteins and assigned new designations (https://www.bpprc.org/) (2, 27).

B. thuringiensis expression plasmids, bulked up in E. coli, were transformed by electroporation into an atoxigenic B. thuringiensis expression strain for protein production (43). B. thuringiensis expression strains were selected on Luria agar (LA) plates with 5 µg/µl chloramphenicol and colonies picked for protein expression in C2 liquid culture with 5 µg/µl chloramphenicol. Cultures were incubated at 28°C for 3 to 4 days in 1× C2 medium [3.11 g KH_2_PO_4_, 4.66 g K_2_HPO_4_, 2.0 g peptone, 2.0 g yeast extract, 2.0 g (NH_4_)_2_SO_4_, 5.0 g casamino acid, 10.0 g glucose dissolved in 900 ml ddH_2_O, and 100 ml 10× C2 salts composed of 3.0 g MgSO_4_·7H_2_O, 1.0 g CaCl_2_·2H_2_O, 0.58 g MnCl_2_·4H_2_O, 0.050 g ZnCl_2_·7H_2_O, 0.050 g CuSO_4_·5H_2_O, and 0.005 g FeSO_4_·7H_2_O in 1 liter] and assessed for crystal formation and sporulation. Protein crystals and spores (CSP) were harvested by centrifugation at 8,000 × *g*; the supernatant was discarded and washed in 10 mM Tris-HCl, pH 8, 0.005% Triton X-100, and 0.1 mM EDTA (pH 8) (TX); and the cell pellet was resuspended in TX at 1/10 the volume of the original culture and stored at 4°C. The insoluble protein in the washed CSPs was solubilized by resuspending the washed cell pellet in 25 mM carbonate buffer (pH 10.0) and 5 mM dithiothreitol (DTT) in 1/10 the original volume followed by centrifugation at 8,000 × *g* to pellet any remaining insoluble debris. The supernatant was collected and stored at 4°C. Cry75Aa protein quantification for CSPs or solubilized CSP was determined by spot densitometry analysis of SDS-PAGE on a Bio-Rad Gel Doc EZ system.

The genes encoding the TIC proteins were cloned into a pET containing an in-frame C-terminal His tag and E. coli expression vector and transformed into E. coli Bl21 cells for protein production. Cells were grown in auto-induction media at 37°C, and the C-terminal His-tag protein was purified using batch nickel chromatography and buffer exchanged into 25 mM carbonate buffer (pH 10) and 5 mM DTT. Proteins estimated to be greater than 90% pure (by SDS-PAGE) were quantified by Bradford assay and stored at −80°C.

### WCR diet overlay bioassay.

For each insect bioassay, WCR eggs were stored at 10°C, 60% rH, and 0:24-h light:dark cycle. Eggs were incubated for 13 d at 25°C, 70% rH, and 0:24-h light:dark cycle. On day 13, eggs were washed from soil and surface disinfested (44) Eggs were incubated overnight for hatch. Two hundred microliters WCR artificial diet (44) was dispensed per well in a 96-well plate, cooled in a laminar flow hood, bagged, and stored at 4°C. On the day of egg hatch (day 14), proteins were removed from the −80°C and thawed by placing on wet ice while sample dilutions were prepared. Prior to diet overlay, a solution containing 4 antibiotics was mixed with the samples to achieve a final concentration of 2.6 µg/ml ciprofloxacin, 4.0 µg/ml colistin, 4.0 µg/ml tobramycin, and 5.0 µg/ml amphotericin B. Samples were arrayed in a 96-deep-well block before overlaying on insect diet. Proteins were mixed thoroughly, and 20 µl of sample was overlaid onto diet per well. Each sample per treatment consisted of 3 replicate columns with 8 wells/column (*n* = 24). Plates were dried in the laminar flow hood before adding one WCR neonate per well with a fine paintbrush. Plates were sealed with VWR silicone adhesive film and ventilated using an “000” insect pin. Bioassay plates were incubated at a 27°C and 70% rH in complete darkness for 6 days and were scored for mortality and instar/stunting as described below. Data were analyzed using JMP 12 statistical software (SAS Institute).

### Colorado potato beetle diet bioassay overlay.

Colorado potato beetle (CPB) (Leptinotarsa decemlineata) egg masses were held at 25°C until needed and then washed in a 1% bleach and 0.1% liquid soap solution. Eggs were placed on Kimwipes in hatch tubs and incubated at 27°C for 4 days or until eggs hatched. Artificial diet, CPB diet product, catalog no. F9380B (Frontier Scientific Services), was prepared according to the manufacturer’s instructions. Samples were arrayed in a 96-deep-well block before overlaying on insect diet. Proteins were mixed thoroughly, and 20 µl of sample was overlaid onto diet per well. Each sample per treatment consisted of 3 replicate columns with 8 wells/column (*n* = 24). Neonates were manually infested with one insect per well in a 96-well diet bioassay plate previously overlaid with protein test samples or buffer/water control samples and air-dried. Plates were sealed with preperforated heat seals and incubated at 27°C incubator for 5 days. Column-wise mortality and stunting were evaluated at the end of the assay as described below.

### Southern corn rootworm diet bioassay overlay.

Southern corn rootworm (SCR) (Diabrotica undecimpunctata howardi) eggs were stored at 10°C. Eggs were separated from soil using clean 30- and 60-mesh sieves. Soil was washed through the sieves with a high-pressure water sprayer, and the clean eggs were collected in a 250-ml beaker. Eggs were sanitized with a 1% bleach solution for 1 min and allowed to settle. Floating debris and dead eggs were decanted followed by a sterile water rinse and decanting and repeated three times. Eggs were washed in 10% formalin and a 0.1% liquid soap solution for 3 min and allowed to settle. Floating debris and dead eggs were decanted followed by a sterile water rinse and decanted; this was repeated three times. SCR eggs were suspended in 0.19% Serva agar solution with a ratio of 1 egg to 20 μl agar and dispensed through a multiple-channel pipettor onto blotting paper. The blotting paper was placed on top of a blank 96-well assay plate and incubated at 25°C with 60 to 70% humidity until hatching. Samples were arrayed in a 96-deep-well block before overlaying on insect diet. Proteins were mixed thoroughly, and 20 µl of sample was overlaid onto diet per well. Each sample per treatment consisted of 3 replicate columns with 8 wells/column (*n* = 24). Neonates were knocked down into a 96-well diet bioassay plate previously overlaid and air-dried with the protein test samples or buffer/water control samples. The infested bioassay plates were incubated at 25°C for 5 days. Assays were scored for mortality and stunting in a column-wise manner as described below.

### Diet bioassay scoring method.

Scoring for all diet assays was completed in a column-wise manner. Sample columns were visually compared to the control H_2_O column individually. Stunting scores were determined based on the visual difference in size between the sample column and the H_2_O column. The stunting score was rated on a 0 to 3 scale. A stunting score of 0, 1, 2, or 3 is defined as less than a <25%, 25 to 50%, 50 to 75%, or >75% difference in size between the sample column and the H_2_O column, respectively. Mortality per well was determined if all larvae within a well were dead. Percent mortality was the number of wells with dead larvae from the total 24 wells. The bioassay was discarded if less than 70% of wells/concentration were infested or with more than 15% contamination. Neonates from a single column of wells from the H_2_O control were collected and weighed at assay completion to confirm control insects met a minimum weight.

### Transgenic plant construction.

*In planta* expression was carried out using both the full-length Mpp75Aa and the mature form of Mpp75Aa (mMpp75Aa; membrane-transiting signal peptide removed) proteins. Codons of the three Mpp75Aas were redesigned for expression in monocots with a GC content of 59%. Mpp75Aas were expressed in a proof-of-concept expression cassette with a high constitutive expression pattern. Cassettes contained a CaMV35S promoter with a duplicated enhancer (45), a 5′ leader sequence from the wheat major chlorophyll *a*/*b*-binding gene (46), an intron from the rice actin 1 gene inserted between the leader and coding sequence (47), and a 3′ untranscribed region (UTR) from the wheat 17-kDa heat shock protein gene (48). In the second set of constructs, a plastid-targeting signal peptide from Setaria italica granule-bound starch synthase (49) was fused in-frame with the coding sequences of the mMpp75Aa homologs. All cassettes were cloned into binary vectors containing a CP4_EPSPS selection cassette providing glyphosate resistance. These six cassettes were transformed into the maize LH244 genotype. Single-copy R0 events were selected based on molecular assays and grown to produce hybrid F1 seed by transferring pollen from transgenic R0 events to a 93IDI3 donor ear. F1 seeds were germinated and were sprayed with glyphosate after a week to eliminate transgene-negative segregants.

### Growth chamber root protection assay.

Seeds from different maize lines were individually germinated or transplanted into 8-in. pots containing Berger BM6 soil in growth chamber at 25°C day/21°C night, 16:8-h light:dark cycle, 50% humidity, and 650 luminescence after surviving a glyphosate application. Protein expression in all plants was confirmed through tissue sampling and enzyme-linked immunosorbent assay (ELISA). WCR eggs from various colonies were incubated at 25°C and 60% rH in total darkness for 13 days until they were about to hatch. At approximately V4, six plants from each maize line were infested with 2,000 WCR eggs per plant. Twenty-four days after infestation, roots were removed from pots and evaluated for larval feeding using the 0 to 3 node injury scale (NIS) (50). Nontransgenic corn (negative control) and current commercial rootworm-protected corn hybrids (SmartStax, MON88017, or Herculex RW) were planted and infected with the same number of rootworm eggs as controls. An NIS score of less than 0.25 is considered a threshold for advancement for further testing.

### Field trial root protection assay.

Ten locations across the Corn Belt were chosen in 2017 due to high WCR pressure the previous season. Three individually randomized blocks of rows of transgenic lines were planted as replications in each location in late April through mid-May. Usual agronomic practices except application of insecticides were followed for each location. Tassels were removed at VT/T1 stages in mid- to late July. Ten plants from each row were randomly selected and dug for assessment of corn rootworm damage using the 0 to 3 NIS. The highest pressure was observed in the trials from Colesburg, Iowa, and Fairbank, Iowa, where the mean NIS of ≥2.0 on negative controls was observed.

### Adult beetle emergence trial.

Six replications of isoline and mMpp75Aa1-expressing maize (with relatively low and high expression) were planted in Nebraska at two locations (Leigh and Shelby) with putative high WCR pressure in late April to early May 2017. Normal agronomic practices for the area were followed, and no insecticides were applied at any time. Leaf tissue was collected from each plant at ∼V2 stage and tested with PCR for genes of interest. Plants that did not test as expected were manually removed, and the remaining plants were thinned to approximately the same density. Tents (3 m by 3 m by 2 m) were erected over plants for each maize line prior to the onset of adult beetle emergence. Adults in each tent were collected every 5 to 7 days until no beetles were observed to emerge for 10 days. WCR beetles were identified and counted for each collection. The percent reduction in adult emergence compared to the control was determined as described previously (29).

### Larval recovery assay.

A single kernel of maize seed from mMpp75Aa1, Herculex RW, or wild-type control maize was planted into 2.36-liter containers (U.S. Plastics, Lima, OH) filled with Berger BM6 potting mix. Each pot had a hole of ∼6.35 mm in diameter with a 25 mm by 25-mm, 530-micron Amber Lumite mesh screen (BioQuip Products, Rancho Dominguez, CA) glued over to facilitate proper drainage. Ten plants per line were maintained in a growth chamber under conditions for growing maize (25°C, 16:8-h light:dark cycle, 50% relative humidity) and watered and fertilized (Peter’s 20-20-20 fertilizer at 300 to 360 ppm nitrogen) as necessary. Each container was infested with 30 neonates from nondiapausing-susceptible (Crop Characteristics) or Cry34Ab1/Cry35Ab1-resistant WCR (8, 51) when plants reached approximately the V4 growth stage. Larvae were left to feed for 10 days, after which root and soil were removed from pots and placed on a Berlese funnel for 3 days. Larvae were collected into 50% ethanol, with counts and instars recorded following collection. Individual larvae were given a numerical value based on mortality (0) or instar stages (1st, 2nd, and 3rd). Adopted from a method described previously (52), a larval instar score (LIS) was used to quantify the average larval development of the 30 neonate larvae infested on each maize plant, calculated as (0 × no. of mortality + 1 × no. of 1st instar + 2 × no. of 2nd instar + 3 × no. of 3rd instar)/30. Between-treatment comparisons were based on the statistical analysis of all observed LIS with the statistical model *y_ijk_* = μ + *T_i_* + *C_j_* + (*TC*)*_ij_* + *e_ijk_*, where μ is the overall mean across all combinations of maize lines and colonies in all replicates, *T_i_* is the diet treatment effect from maize line *i*, *C_j_* is the rootworm colony effect from colony *j*, (*TC*)*_ij_* is the interaction effect between maize line *i* and colony *j*, and *e_ijk_* is the residual effect for replication *k*. A square root transformation was applied to account for the count nature of the data. Analysis was performed with SAS version 9.4.

### Data availability.

The sequences for the 3 genes and protein translations are available in GenBank. The accession number for the TIC3670 (Cry75Aa1) gene is MF490291.1, and that for the protein is ASY04853.1. The accession number for the TIC3669 (Cry75Aa2) gene is MF490290.1, and that for the protein is ASY04852.1. The accession number for the TIC3668 (Cry75Aa3) gene is MF490289.1, and that for the protein is ASY04851.1. The protein sequences and new nomenclature are available at the Bacterial Pesticidal Protein Resource Center (BPPRC) (https://www.bpprc.org/). Cry75Aa1 has been renamed Mpp75Aa1, Cry75aa2 has been renamed Mpp75Aa2, and Cry75Aa3 has been renamed Mpp75Aa3.
